# Lipid Droplets in the Pathogenesis of Hereditary Spastic Paraplegia

**DOI:** 10.3389/fmolb.2021.673977

**Published:** 2021-05-10

**Authors:** Nimesha Tadepalle, Elena I. Rugarli

**Affiliations:** ^1^ Molecular and Cell Biology Laboratory, Salk Institute of Biological Sciences, La Jolla, CA, United States; ^2^ Institute for Genetics, University of Cologne, Cologne, Germany; ^3^ Cologne Excellence Cluster on Cellular Stress Responses in Aging-Associated Diseases (CECAD), Cologne, Germany; ^4^ Center for Molecular Medicine (CMMC),Cologne, Germany

**Keywords:** hereditary spastic paraplegia, lipid droplet, lipid metabolism, endoplasmic reticulum, spastin

## Abstract

Hereditary spastic paraplegias (HSPs) are genetically heterogeneous conditions caused by the progressive dying back of the longest axons in the central nervous system, the corticospinal axons. A wealth of data in the last decade has unraveled disturbances of lipid droplet (LD) biogenesis, maturation, turnover and contact sites in cellular and animal models with perturbed expression and function of HSP proteins. As ubiquitous organelles that segregate neutral lipid into a phospholipid monolayer, LDs are at the cross-road of several processes including lipid metabolism and trafficking, energy homeostasis, and stress signaling cascades. However, their role in brain cells, especially in neurons remains enigmatic. Here, we review experimental findings linking LD abnormalities to defective function of proteins encoded by HSP genes, and discuss arising questions in the context of the pathogenesis of HSP.

## Introduction

### Hereditary Spastic Paraplegia

In humans, voluntary and fine motor movements are controlled by the corticospinal tract, which is composed of more than a million axons of the pyramidal motor neurons (Welniarz et al., 2017). The majority of these axons travel through the internal capsule and the brainstem, decussate in the caudal medulla, and descend in the lateral funiculus of the contralateral spinal cord to make mono-synaptic contacts with spinal motor neurons in the ventral horn (contralateral corticospinal tract). A small portion of corticospinal axons descent unilaterally to form the ipsilateral corticospinal tract. Progressive degeneration of the distal corticospinal axons causes weakness and spasticity (rigidity) of the lower limb. These symptoms define pure forms of hereditary spastic paraplegia, a neurological condition with a prevalence of 1–10 per 100,000 individuals (Shribman et al., 2019; Lallemant-Dudek and Durr, 2020). Complicated HSP is characterized by the association of other clinical symptoms, including ataxia, intellectual disability, cognitive impairment, epilepsy, amyotrophy, and peripheral neuropathy (Blackstone, 2018b; Shribman et al., 2019; Lallemant-Dudek and Durr, 2020). This clinical complexity is reflected in a vast genetic heterogeneity of HSP, with over 80 genes classified in the OMIM database (www.omim.org) so far. HSP can be inherited as autosomal dominant, autosomal recessive or X-linked (Schule and Schols, 2011). The age of onset spans from childhood to late life. So far, apart from treatments to reduce spasticity, there is no available strategy to block the progression of the symptoms.

Corticospinal axons projecting to the lumbar spinal motoneurons can reach the outstanding length of 1 m in adult humans, contain 99% of the neuronal cytosol, and strongly depend on efficient trafficking mechanisms to transport macromolecules and organelles at sites very distant from the oma. The extreme polarization of cortical motor neurons makes them vulnerable to even minor disturbances of a plethora of pathways. Disruption or abnormalities of endosomal trafficking and dynamics, mitochondrial quality control, endoplasmic reticulum (ER) morphogenesis, cargo-mediated transport on the microtubules, lysosomal function, and lipid metabolism play a role in the pathogenesis of HSP (Blackstone et al., 2011; Rugarli and Langer, 2012; Allison et al., 2017; Blackstone, 2018a; Darios et al., 2020). Here, we focus on the intriguing connection between several HSP proteins and the biology of lipid droplets, and discuss the possible role of LD dysfunction in the pathogenesis of the disease.

### Lipid Droplets

Organelles consist of an aqueous environment harboring various macromolecules that are surrounded by a phospholipid bilayer, leading to the compartmentalization of specific biochemical reactions occurring in a cell. Phase separation of a hydrophobic lipid core from the aqueous environment of the cell by a phospholipid monolayer makes LDs unique. LDs are evolutionary conserved, ubiquitous organelles found in prokaryotes, as well as in unicellular and higher eukaryotes (Murphy, 2001; Alvarez and Steinbuchel, 2002; Fujimoto and Parton, 2011; Ohsaki et al., 2014). The neutral lipid core of LDs consists of triacylglycerols (TAGs), sterol esters (SE) and rarely retinyl esters (Brasaemle, 2007; Fujimoto and Parton, 2011; Walther and Farese, 2012; Barbosa and Siniossoglou, 2017; Walther et al., 2017). The LD surface is composed of a different combination of phospholipids. In mammalian cells, the phospholipid monolayer of LDs is highly enriched in phosphatidylcholine (PC), followed by phosphatidylethanolamine (PE) (Bartz et al., 2007). In yeast, on the other hand, phosphatidylinositol (PI) is the second most abundant phospholipid species on LDs, after PC (Grillitsch et al., 2011; Ohsaki et al., 2014; Wang, 2015). A compendium of proteins coats the LD surface, and contributes to the function of this organelle (Kory et al., 2016; Bersuker et al., 2018).

LDs play critical roles by storing fatty acids (FAs), which can then be delivered to mitochondria or peroxisomes for β-oxidation, or be utilized to synthesize new membranes and mediate lipid signaling. Therefore, LDs are key organelles in energy homeostasis, lipid metabolism, and signal transduction. Furthermore, LDs have been implicated in regulating ER stress and in clearing of protein aggregates (Walther and Farese, 2009; Fujimoto and Parton, 2011; Walther and Farese, 2012; Walther et al., 2017; Olzmann and Carvalho, 2019; Roberts and Olzmann, 2020). LD dysfunction is the hallmark of several diseases, such as obesity, diabetes, hepatic steatosis, cardiovascular disease and cancer (Krahmer et al., 2013; Welte, 2015; Gluchowski et al., 2017; Goldberg et al., 2018; Pennetta and Welte, 2018; Cruz et al., 2020; Seebacher et al., 2020). Recently, motor neuron diseases have become subjects of investigation by LD experts due to aberrant LD biology observed in neurodegenerative diseases like amyotrophic lateral sclerosis, Huntington’s disease and HSP (Farmer et al., 2020). In the following sections, we review the experimental evidence that links the function of HSP proteins to every step of the LD life cycle (Table 1), and discuss the possible impact of these observations on the pathogenesis of HSP.

**TABLE 1:** Identified spastic paraplegia (SPG) genes involved in LD metabolism.

Disease/gene	Protein name	Known molecular function	LD phenotype	Model system
SPG3A/*ATL1*	Atlastin-1	ER morphogen	Increase in LD number	Astrocytes
			Decrease in LD size	Astrocytes, worms, flies
			Abnormal LD distribution	Worms, flies
SPG4/*SPAST*	Spastin (M1 and M87 isoforms)	ER morphogen Microtubule severing	Increase in LD number	MEFs, NSC34 cells
			Increase in LD biogenesis	MEFs
			Decrease in LD size	Worms, flies
			Abnormal LD distribution	Zebrafish, MEFs
			LD-peroxisome contacts	Hela cells
SPG11/*KIAA1840*	Spatacsin	Autophagic lysosomal turnover	Lysosomal turnover of lipids	MEFs, neurons
SPG17/*BSCL2*	Seipin	LD biogenesis	LD biogenesis, emergence, maturation	Yeast, A431, *Drosophila* S2, SUM 159, and Hela cells
			LD detachment	A431 cells
SPG18/*ERLIN2*	ERLIN2	ER-associated degradation	LD accumulation	Hela cells
SPG20/*SPART*	Spartin	Endosomal trafficking	LD turnover	HEK 293T, Hela cells
			LD accumulation	HEK 293T, Adipose tissue, MEFs
SPG31/*REEP1*	REEP1	ER morphogenesis	Decrease in LD number and size	MEFs
			Decrease in LD number	Neurons, adipocytes
SPG54/*DDHD2*	Phospholipase A1	Phospholipase, TAG lipase in the brain	LD accumulation	Mouse brain and spinal cord
			Peroxidized lipid accumulation	MEFs
SPG62/*ERLIN1*	ERLIN1	ER-associated degradation	LD accumulation	Hela cells
SPG73/*CPT1C*	CPT1C	Binding malonyl-CoA	Decrease in LD number	Neurons
*BICD2*	BICD2	Dynein-mediated motility	LD movement	Fly embryos

## Disturbances of LD Biogenesis in HSP Pathogenesis

### HSP Proteins and the Shaping of the Tubular ER

In eukaryotic cells, LDs are assembled at the ER through a series of sequential steps beginning with FA activation by esterification with coenzyme A, followed by synthesis of TAG or SE by enzymes residing in the ER. LD biogenesis begins when neutral lipids get sequestered between the two leaflets of the ER membrane, leading to the bulging of a lens-like structure, in a process that is known as the LD nucleation. With increasing neutral lipid synthesis, this lens structure expands to form a nascent LD, which further grows in a mature LD. The latter bulges toward the cytosolic side of the ER, and can ultimately detach to form a separate entity. Several excellent reviews have described in detail various steps of LD biogenesis (Walther and Farese, 2009, 2012; Pol et al., 2014; Henne et al., 2020).

**FIGURE 1:**
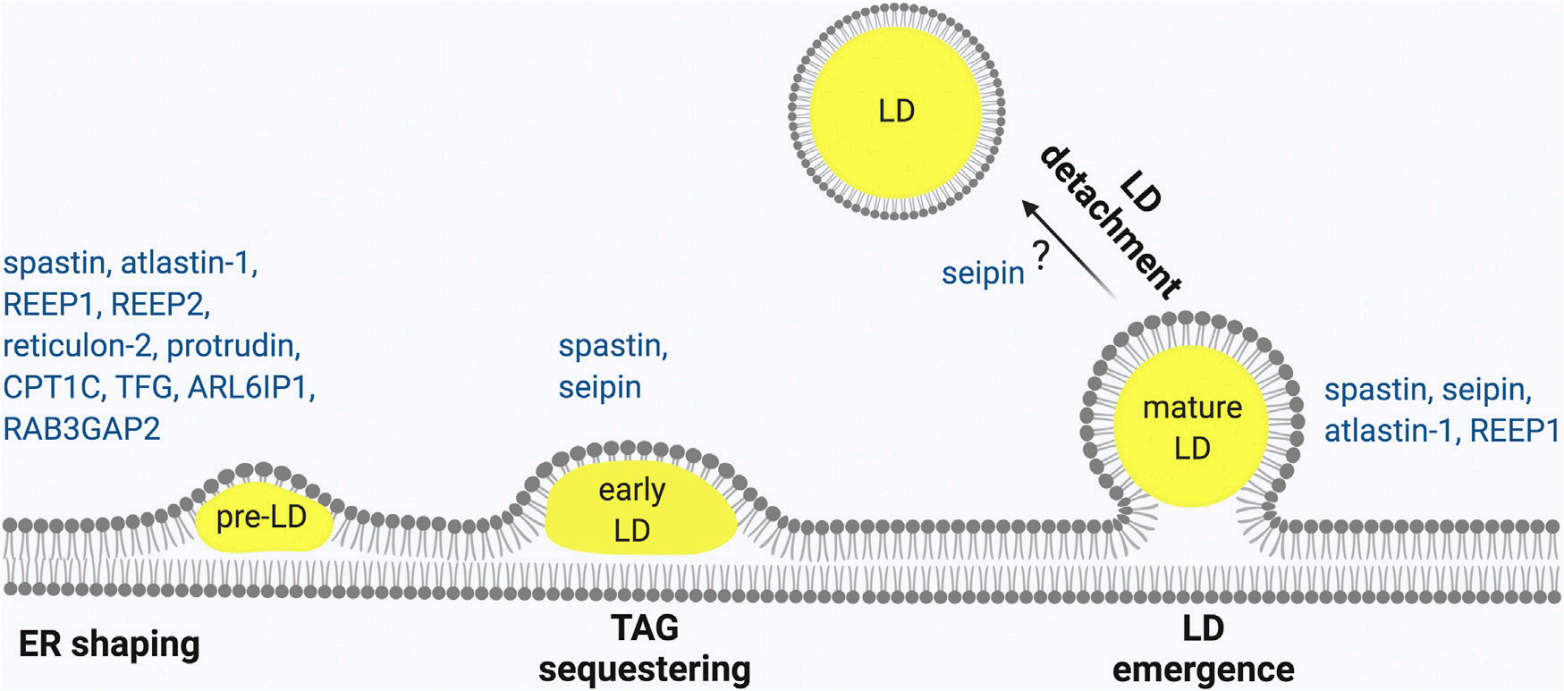
HSP proteins involved in different steps of LD biogenesis. LD biogenesis progress occurring at the ER as shown from left to right. (1) ER morphogens regulate membrane curvature required to initiate LD biogenesis. (2) Neutral lipid accumulates between the two leaflets of the ER to form nascent LDs that (3) continue to expand to early LDs (4) and finally lead to mature LDs that (5) may undergo detachment from the ER. HSP-associated proteins involved in each of these steps are indicated. Made with Biorender.com.

The ER is composed of different compartments, the nuclear envelope, the ER sheets and the ER tubules, however LD biogenesis mainly occurs at ER tubules, at least in mammalian cells (Kassan et al., 2013; Santinho et al., 2020). One major difference between ER tubules and sheets is that ER sheets are mainly flat, while ER tubules are highly curved. Membrane curvature at the ER tubules decreases the critical concentration of TAG necessary for LD nucleation (Santinho et al., 2020). While the rough ER is only present in neuronal Soma, the tubular ER extends into axons, where it forms a long narrow continuous system (Terasaki, 2018; Öztürk et al., 2020). Maintenance of the tubular ER compartment is crucial for axonal health, as demonstrated by the involvement of several proteins that shape the tubular ER in axonopathies (Hubner and Kurth, 2014). Remarkably, genetic experiments of overexpression or depletion of these HSP proteins in various model organisms has revealed LD abnormalities, pointing to an intimate connection between ER tubule shaping and the formation of LDs (Figure 1).

Atlastin-1, −2 and −3 belong to the dynamin family of GTPases and play a well-established role in homotypic ER membrane fusion (Hu et al., 2009; Orso et al., 2009; Kim et al., 2017). Atlastins localize to three-way junctions at the ER tubules and target the ER membrane using a long hydrophobic domain that insert in the membrane as a hairpin (Park et al., 2010). Mutations in *ATL1* are responsible for HSP type 3 (Sauter et al., 2004), while *ATL3* is mutated in hereditary sensory neuropathy (Kornak et al., 2014). Cells lacking or expressing dominant negative forms of atlastins show fragmented and unbranched ER tubules (Rismanchi et al., 2008; Hu et al., 2009; Wang et al., 2016b; Zhao et al., 2016; Behrendt et al., 2019). ER morphology defects with less three-way junctions and fragmented tubules have also been observed in flies lacking atlastin, while atlastin antibodies impaired ER network formation *in vitro* using a membrane fraction from *Xenopus* oocytes (Hu et al., 2009; Orso et al., 2009).

The *C. elegans* orthologue of atlastin-1 (ATLN-1) was found as a hit in a genetic screen aimed at identifying mutants with less LDs. Worms depleted or expressing mutant forms of ATLN-1 displayed smaller LDs that were abnormally distributed as compared to wildtype worms (Klemm et al., 2013). A similar phenotype was observed in *Drosophila*, where downregulation of the atlastin orthologue led to reduced accumulation of LDs and TAGs in the fat body, rendering fly larvae more susceptible to starvation. Furthermore, the membrane fusion activity of atlastin-1 was responsible for mediating this LD phenotype (Klemm et al., 2013). Smaller numerous LDs were recently observed in astrocytes derived from human pluripotent stem cells carrying *ATL1* disease mutations (Mou et al., 2020).

Atlastins interact with members of the reticulon and the REEP/DP1/Yop1 protein families, which contain a reticulon-homology domain that target them at the ER tubules (Shibata et al., 2010). This domain consists of two hydrophobic regions, each 28–36 amino acids long, which are too long to span the membrane once and instead form a hairpin structure, embedded as a wedge and imparting curvature to the membrane. Consistently, in absence of reticulons and REEPs, the tubular ER is reduced (Shibata et al., 2010; Wang et al., 2016b; Yalcin et al., 2017). *REEP1* is the causative gene of SPG31, a relatively common autosomal dominant HSP (Zuchner et al., 2006). A closely related gene, *REEP2,* is mutated in SPG72 (Esteves et al., 2014), while mutations in *RTN2*, encoding reticulon-2, lead to HSP type 12 (Montenegro et al., 2012). *Reep1* null mice exhibit a progressive motor phenotype, without obvious degeneration of the corticospinal axons. Surprisingly, they also showed a mild lipoatrophy, consistent with a defect in LD formation in adipose tissue (Renvoise et al., 2016). Moreover, mouse embryonic fibroblasts (MEFs) and neurons isolated from these mice showed fewer and smaller LDs (Renvoise et al., 2016). In normal condition, REEP1 is never found on LDs, however when HSP-associated mutant variants of REEP1 were expressed in mammalian cells they were mistargeted to LDs. Notably, atlastin-1 and REEP1 cooperate in controlling LD size. In fact, concomitant overexpression of atlastin-1 and REEP1 in mammalian cells leads to the formation of bigger LDs (Falk et al., 2014).

Atlastin-1 also interacts with CPT1C, the neuronal isoform of carnitine palmitoyl-transferase (*CPT1C*), suggesting a role of this protein in ER morphogenesis (Rinaldi et al., 2015). Mutations in *CPT1C* were recently identified to cause a pure form of autosomal dominant HSP (SPG73) (Rinaldi et al., 2015). This protein does not catalyze the transfer of fatty acyl to carnitine, but rather binds malonyl-CoA and may sense lipid availability (Wolfgang et al., 2006). Interestingly, knock-out mice for CPT1C display reduced body weight and food intake, but are prone to obesity when fed a high fat diet (Wolfgang et al., 2006). Neurons isolated from CPT1C null mice possess fewer LDs (Rinaldi et al., 2015). The expression of mutant forms of CPT1C involved in HSP reduce LD number and size in a dominant negative manner (Rinaldi et al., 2015).

In conclusion, ER-resident morphogenesis proteins involved in HSP interact with one another and play essential roles in regulating LD size and formation (Figure 1). Consistently, alteration of the ratio of sheets vs. tubules by overexpression of atlastin-1, reticulon-4 or REEP-5, which induce ER tubulation, led to an increase in LD nucleation (Santinho et al., 2020), indicating that proteins involved in ensuring bending and curvature of the ER tubules assist in LD emergence (Roberts and Olzmann, 2020). This conclusion agrees with the *in vivo* phenotypes of atlastin-1, and REEP1 deficient models, which are all characterized by a defect in LD biogenesis. Other HSP proteins, such as TFG, ZFYVE27, RAB3GAP2, and ARL6IP1, also contribute to ER shaping and may therefore affect LD biogenesis (Hubner and Kurth, 2014).

### Spastin M1, a Microtubule-Severing Protein, Is Recruited to LDs

Compelling links to LD function have been uncovered for spastin, the product of *SPAST*, which is the most commonly mutated autosomal dominant HSP gene (Hazan et al., 1999). Spastin is a well-characterized microtubule-severing protein (Errico et al., 2002; Evans et al., 2005; Roll-Mecak and Vale, 2008). Spastin belongs to the AAA (ATPases Associated with various cellular Activities) family of ATPases (Sauer and Baker, 2011), and uses the energy derived from ATP hydrolysis to destabilize the microtubule lattice leading ultimately to the internal breaking (severing) of the tracks (Roll-Mecak and Vale, 2008). Spastin also harbors a MIT (microtubule-interacting and trafficking) domain, which can recruit the ESCRTIII proteins IST1 and CHMP1B (Reid et al., 2005; Allison et al., 2013).

In mammalian cells, four isoforms of spastin are produced by alternative splicing of exon 4 and dual initiation of translation (Claudiani et al., 2005). When spastin translation starts at the first in frame AUG in exon 1, the resulting protein (spastin M1) comprises a stretch of 86 amino acid at the N-terminus, which contains a hydrophobic region and targets the protein at the ER (Connell et al., 2009; Park et al., 2010). When carrying mutations in the AAA domain trapping the protein on the microtubules, spastin M1 specifically bundles perinuclear microtubules associated with the ER, which are more resistant to depolymerization (Plaud et al., 2018). In contrast, spastin isoforms starting at the methionine M87 localize to the cytosol and endosomal compartments (Connell et al., 2009; Plaud et al., 2018). Spastin M87 is abundant in all cells and tissues, and has been implicated in several cellular processes, such as endosomal tubulation (Allison et al., 2013), midbody abscission and cytokinesis (Connell et al., 2009), mitotic spindle disassembly (Zhang et al., 2007; Vietri et al., 2015), and lysosomal function (Allison et al., 2019). Spastin M87 display efficient microtubule-severing activity and its overexpression disrupts the microtubule cytoskeleton (Errico et al., 2002).

Despite the fact that the expression of spastin M1 is kept low by both transcriptional and translational control mechanisms (Claudiani et al., 2005; Mancuso and Rugarli, 2008), the synthesis of M1 isoforms is evolutionary conserved not only in mammals, but also in zebrafish (Arribat et al., 2020). Furthermore, spastin M1 interacts with other HSP proteins and ER morphogens, including atlastin-1, REEP1, REEP2, reticulon-1, and protrudin (Mannan et al., 2006a; Mannan et al., 2006b; Sanderson et al., 2006; Park et al., 2010; Esteves et al., 2014), suggesting a pathogenic role of this isoform and a contribution to ER shaping. Spastin M1 behaves as a class I LD protein, i.e. proteins that are inserted in the ER membrane and can laterally diffuse from the outer leaflet of the ER to the LD phospholipid monolayer (Kory et al., 2016; Roberts and Olzmann, 2020). Consistently, the hydrophobic domain of spastin M1 adopts a hairpin conformation and mediates targeting of the protein from the ER to nascent and mature LDs when cells are loaded by oleic acids (Park et al., 2010; Papadopoulos et al., 2015; Chang et al., 2019).

What is the exact role of spastin at the LDs? Answering this question is complicated by the presence of the different spastin isoforms and the fact that manipulation of spastin levels interferes with microtubule dynamics. However, modeling spastin deficiency *in vivo* in several organisms has unraveled LD biogenesis defects. The *Drosophila* Dspastin exists as a unique form with an extended N-terminus. Loss of function of Dspastin obtained by RNA interference or by expressing a dominant-negative mutant reduced the number and size of LDs in the fat body, the skeletal muscle and the peripheral nerves (Papadopoulos et al., 2015). Decreased accumulation of LDs was also observed in the *C. elegan*s gut, upon deletion or depletion of the spastin orthologue (Papadopoulos et al., 2015), although in the worm only a short spastin variant is described. In the zebrafish, a knock-out of all spastin isoforms by CRISPR-Cas9 gene editing led to a metabolic phenotype with reduced mitochondrial respiration and glycolysis, lipid accumulation around the swim bladder and ER abnormalities in the skeletal muscle (Arribat et al., 2020).

In apparent contrast with these findings *in vivo*, cultured cells lacking spastin showed an increased number of LDs accompanied by enhanced synthesis of TAGs, which were rescued by re-expressing spastin M1 and to a lesser extent spastin M87 (Tadepalle et al., 2020). This phenotype was particularly prominent upon severe starvation, a condition that stimulates LD biogenesis (Rambold et al., 2015). An increased number of smaller LDs was also observed in cells derived from spastin KO fish embryos (Arribat et al., 2020). Overexpression of spastin M1 on the other hand induced the appearance of large, but fewer LDs (Papadopoulos et al., 2015; Arribat et al., 2020). Since spastin M1 localizes to nascent LDs (Chang et al., 2019; Tadepalle et al., 2020), an explanation for these findings could be in a specific role of spastin M1-containing hexamers that are anchored to the ER in shaping the ER and the microtubule network at sites where LDs are formed, limiting LD nucleation but promoting their maturation. In line with this hypothesis, a synthetic peptide comprising the spastin M1 membrane-spanning sequence promoted phospholipid transbilayer movement (flip-flop) in artificial membranes (Nakao et al., 2016). Since reducing phospholipids at the cytoplasmic leaflet of the ER membrane could hamper LD emergence (Chorlay et al., 2019), it is possible that absence of spastin favors LD emergence. Lack of microtubule-severing could instead affect the ability of LDs to grow.

Different metabolic conditions and nutrient availability, as well as a proliferative vs. a post-mitotic status can underlie the distinct phenotypes *in vivo* and *in vitro* upon spastin depletion. It is also conceivable that the LD phenotype observed in cultured cells represents a compensatory mechanism. Spastin-deficient cells did not show abnormal ER shaping and distribution, however they depended on synthesis of TAG to preserve the general morphology of the ER upon starvation (Tadepalle et al., 2020). Similar findings have been shown in yeast cells in which LD formation is hampered (Velázquez et al., 2016). It is possible that increasing LD biogenesis simply expands the ER membrane, which has been shown to alleviate ER stress (Schuck et al., 2009). Consistent with this hypothesis is the observation that zebrafish lacking spastin are more susceptible to ER stress and displayed abnormal ER organization in sarcomeres (Arribat et al., 2020).

Together, these data obtained in various model systems, strongly support a role of spastin as a central regulator of LD behavior, but do not allow to conclusively dissect the role of spastin M1, which binds LDs, in respect to spastin M87, which can affect LDs indirectly by modulating microtubule dynamics. As we discuss in a following chapter, spastin M1 also regulates LD movement, and LD-peroxisome interaction, opening the question as to which of these multiple roles is relevant in the pathogenesis of HSP.

### Seipin-Related Motoneuron Diseases

Seipin is widely recognized as a key molecular player in LD biogenesis [see (Salo et al., 2020) for a recent comprehensive review]. Seipin is an integral ER membrane protein with two transmembrane domains, exposing both N- and C-termini to the cytosol (Lundin et al., 2006). Seipin forms undecamers that localize in ER tubules and are highly mobile, but arrest at ER-LD contact sites (Yan et al., 2018; Santinho et al., 2020). Although LDs can form also in its absence (Santinho et al., 2020), seipin regulates LD assembly in multiple ways, both at early stages and during maturation. Seipin has been implicated in defining the site of LD nucleation, controlling phospholipid metabolism at the ER tubules, and mediating neutral lipids shuttling from the ER to LDs (Salo et al., 2016; Wang et al., 2016a; Yan et al., 2018; Chung et al., 2019). Consistently, lack of seipin in a variety of model systems, ranging from yeast to mammalian cells induces LD abnormalities, with several small LDs and few large LDs, while seipin overexpression efficiently inhibits LD formation (Szymanski et al., 2007; Fei et al., 2008; Fei et al., 2011). Moreover, lack of seipin leads to severe loss of ER-LD contacts (Salo et al., 2016). Thus, seipin functions as gatekeeper at ER-LD contact sites to regulate LD formation with a mechanism that is not completely understood.

Consistent with this role in LD biogenesis, human patients carrying loss-of-function mutations in *BSCL2*, the gene encoding for seipin, suffer from Berardinelli-Seip congenital lipodystrophy (Magre et al., 2001), a severe recessive disease characterized by abnormal fat deposition. In contrast, missense mutations in seipin at residues located in the ER lumen, N88S and S90L, are responsible of dominant forms of HSP (SPG17) and other neurological conditions, such as Silver syndrome, Charcot-Marie-Tooth neuropathy type 2, and distal hereditary motor neuropathy (dHMN) type V (Irobi et al., 2004; Windpassinger et al., 2004). These disorders, which have been dubbed seipinopathies, are all characterized by progressive axonopathy with various involvement of central and peripheral axons (Ito and Suzuki, 2009). From a pathogenic point of view, these missense mutations act via a gain-of-function mechanism, by hampering glycosylation of the protein. When expressed in yeast cells lacking *Fld1*, the seipin orthologue, these mutants are perfectly capably to rescue the LD phenotype (Fei et al., 2008).

What is the pathogenic mechanism of seipinopathies? Expression of seipin N88S and even more of S90L in non-neuronal cells triggers the formation of aggregates, and causes ER stress. Interestingly, the presence of these aggregates appeared to be protective and reduce apoptotic cell death (Ito et al., 2012). Later, aggregates of seipin mutants were shown to colocalize with LDs in cells incubated with oleic acid. Under this condition, cells exhibited reduced ER stress and better survival (Holtta-Vuori et al., 2013). Similarly, lipid loading rescued a swimming defect of a seipinopathy zebrafish model system (Holtta-Vuori et al., 2013). In this case, as in spastin knock-out cells, TAG loading and increased LD biogenesis may be a protective mechanism to prevent ER stress. Abnormal formations, with an ultrastructure reminiscent of LDs, were also observed in neurons of transgenic mice expressing the N88S seipin mutation (Yagi et al., 2011). It is important to stress that all these studies are based on a non-physiological overexpression of mutant seipin, however these data form a compelling case for a dysfunction of the tubular ER in *BSCL2*-related motoneuron diseases.

## HSP Proteins and LD Turnover and Quality Control

**FIGURE 2:**
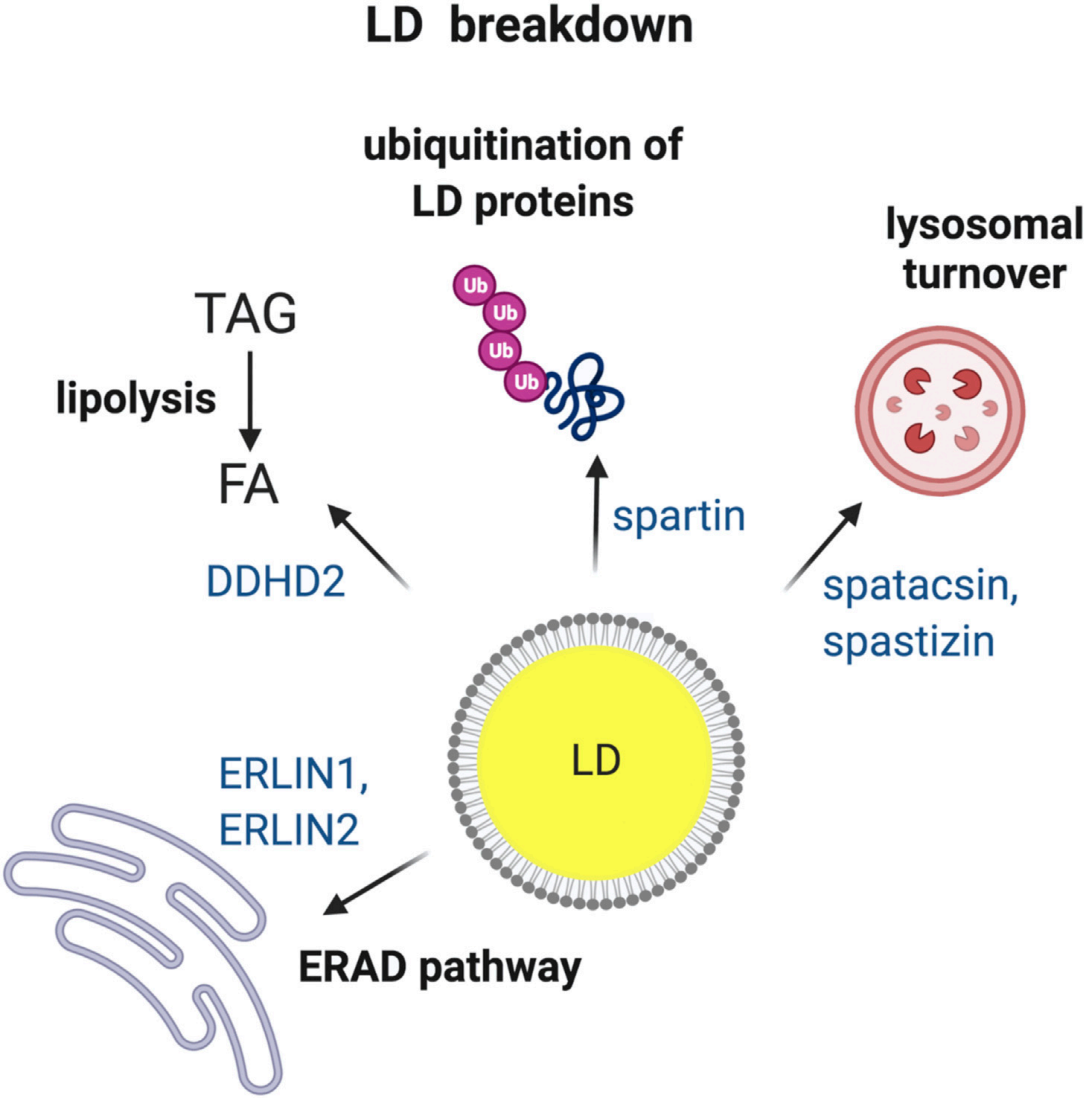
HSP proteins involved in the breakdown of LDs. Turnover of LDs via four different pathways i.e., (1) ER-associated degradation (ERAD); (2) TAG hydrolysis by lipases; (3) ubiquitin-mediated degradation of specific LD proteins and (4) breakdown of LDs by lysosomes via autophagy. HSP-associated proteins involved in each of the different pathways are indicated. Made with Biorender.com.

When cells sense energy deficit, existing LDs can be utilized to provide FAs as fuel. LDs can be broken down by two major pathways: 1) lipolysis, i.e., hydrolysis of TAG or SE by respective enzymes to finally release FA, and 2) autophagy, i.e., engulfment of LDs by lysosomes leading to breakdown of LDs finally releasing FA (Hashemi and Goodman, 2015). Furthermore, LDs proteins can be targeted to degradation by ubiquitin-dependent pathways (Roberts and Olzmann, 2020). Experimental evidence has linked HSP proteins to abnormal turnover of LDs (Figure 2), raising the question of its pathogenic significance.

### DDHD2: A Specific Brain TAG Lipase

The strongest claim for a defect of LD turnover in HSP is linked to mutations in *DDHD2* in recessive SPG54 (Schuurs-Hoeijmakers et al., 2012). SPG54 families display early onset HSP, with thin corpus callosum and an abnormal lipid peak on cerebral proton magnetic resonance spectroscopy (Schuurs-Hoeijmakers et al., 2012). DDHD2 belong to the family of phospholipase A_1_, however later a role as a specific brain TAG lipase has been proposed and confirmed using the recombinant protein (Inloes et al., 2014). Disruption of *Ddhd2* in mice leads to defects in motor coordination and cognition, hallmarks of HSP (Inloes et al., 2014). Remarkably, massive accumulation of TAG and LDs was observed in the brain and spinal cord of these mice*,* while other organs like the adipose tissue and heart remained unaffected (Inloes et al., 2014). HSP-associated disease mutants of DDHD2 failed to hydrolyze TAGs and to repress the LD accumulation in cells loaded with oleic acid (Inloes et al., 2018). Furthermore, mass spectrometric analysis of the LD proteome from brain tissue of mice lacking *Ddhd2* detected enrichment of nervous system specific proteins, including several implicated in neurological conditions. Therefore, accumulation of LDs upon loss of DDHD2 could not only lead to the inability to mobilize fat stores, but also to the sequestration and disruption of the trafficking of key proteins and signaling lipids required for maintaining axonal integrity (Inloes et al., 2018).

Recent data have also implicated DDHD2 in ROS production, irrespective of its lipolysis activity (Maruyama et al., 2018). In contrast to neurons, *Ddhd2* knock out MEFs do not accumulate LDs but show increased ROS, enhanced lipid peroxidation, and mitochondrial dysfunction. This role of DDHD2 would depend on a partial localization of the protein to mitochondria and be specific of peripheral tissues (Maruyama et al., 2018). Since ROS production stimulates the formation of lipids in neurons that are transported to glial cells and deposited in LDs (Liu et al., 2015; Liu et al., 2017; Ioannou et al., 2019), it is possible that several mechanisms are at play in HSP caused by deficiency of DDHD2.

### HSP Proteins and Ubiquitin-dependent Degradation Pathways at LDs

The gene encoding for the protein spartin causes a complicated form of HSP called Troyer syndrome (SPG20) (Patel et al., 2002). The N-terminus of spartin contains a MIT domain that is also found in spastin and trafficking proteins involved in protein degradation through the endo-lysosomal system (Ciccarelli et al., 2003). Spartin is a cytosolic protein that can be recruited to LDs upon treatment with oleic acid, via a C-terminal region that is deleted in Troyer syndrome mutants (Eastman et al., 2009; Edwards et al., 2009; Hooper et al., 2010). Spartin colocalizes with TIP47/perilipin 3 at the LDs and competes with perilipin 2 for binding to LDs (Eastman et al., 2009). Downregulation and overexpression of spartin led to accumulation of LDs in cells (Eastman et al., 2009). Furthermore, *Spg20* knock-out female mice displayed increased LD numbers and alterations in perilipin levels in fat tissues (Renvoise et al., 2012). Spartin interacts with the E3 ubiquitin ligases AIP4 and AIP5 and was found to recruit AIP4 to LDs (Eastman et al., 2009; Hooper et al., 2010). As an adaptor for AIP4, spartin regulates the ubiquitination and degradation of perilipin 2. (Hooper et al., 2010). Together, these data support the hypothesis of a key role of this protein in the turnover of crucial component of the LD proteome, and highlight the general role of LDs as sites for protein degradation (Welte, 2007; Roberts and Olzmann, 2020).

ER-associated degradation (ERAD) has been shown to regulate the turnover of proteins that shuttle from the ER tubules to the LDs (Roberts and Olzmann, 2020). The *ERLIN2* gene is mutated in families with complex recessive forms of HSP (*SPG18*), while *ERLIN1* has been implicated in autosomal recessive cases of pure HSP (*SPG62*) (Novarino et al., 2014) ERLIN1 and ERLIN2 are highly homologous proteins, belonging to the SPFH (stomatin/prohibitin/flotilin/HflK/c) family, which is defined by a module of ∼180–200 amino acids that has been found in some members of the family to bind cholesterol (Browman et al., 2007). The general function of SPFH proteins is to act as scaffolds for both lipids and proteins and define membrane microdomains in different cell compartments (Browman et al., 2007). Consistently, ERLIN1 and ERLIN2 have been first isolated as components of cholesterol-rich domains (lipid rafts) of the ER (Browman et al., 2006), and later shown to bind cholesterol (Huber et al., 2013). The ERLIN complex has established functions in regulating ERAD of the inositol 1,4,5 triphosphate (IP_3_) receptors (Pearce et al., 2007), and of 3-hydroxy-3-methylglutaryl-coenzyme reductase (HMGR), a rate-limiting enzyme for cholesterol synthesis (Jo et al., 2011), by interacting with specific E3 ubiquitin ligases. Moreover, the ERLIN complex associates with the sterol regulatory element binding proteins (SREBPs), Insig and Scap at the ER under cholesterol sufficiency. Upon downregulation of ERLINs, the degradation of Insig1 was accelerated, leading to canonical activation of SREBPs and their target genes, including HMGR. Cells depleted of ERLINs accumulated LDs, which may contain cholesterol esters, since both cholesterol and esterified cholesterol were also increased (Huber et al., 2013). Together, these data strongly suggest that ERLINs define ER subcompartments involved in regulating both Ca^2+^ handling and cholesterol metabolism. It is tempting to speculate that the ERLIN complex may regulate the ERAD of other proteins involved in lipid and LD metabolism.

### HSP Proteins and Autophagy

Lysosomes fuse with autophagosomes to degrade cellular organelles, thus maintenance of a functional lysosomal pool in cells is required for proper functioning of autophagy. Two HSP-associated proteins, spastizin (SPG15) and spatacsin (SPG11), are involved in autophagic lysosome reformation, a pathway that generates new lysosomes (Chang et al., 2014). Mutations in spatacsin and spastizin, leads to the loss of free lysosomes and an accumulation of autolysosomes (Chang et al., 2014). Additionally, cortical neurons lacking spatacsin displayed undigested, accumulated LDs in association with lysosomes (Branchu et al., 2017). In primary mouse fibroblasts, loss of spatacsin impaired the autophagy-induced shuttling of fatty acids derived from membrane phospholipids from lysosomes to LDs. This lipid trafficking defects resulted in a decreased number of LDs not only in fibroblasts, but also in cortical neurons from *Spg11* knock-out mice (Branchu et al., 2017). Spatacsin appears to be involved in clearance of gangliosides (Boutry et al., 2018) at the lysosome, suggesting that LDs in neurons may contain specific lipid class.

Recent data suggest that altered lysosomal function may be a recurrent theme in many HSP forms (Allison et al., 2017). Are defects in LD turnover in neurons, owing to impaired lysosomal function, a common pathway in HSP? Notably, atlastins emerged as novel players in mediating ER-phagy, the selective autophagy of ER tubules (Liang et al., 2018; Chen et al., 2019). One may speculate that impairment of ER-phagy in axons could not only compromise the recycling and the quality control of ER tubules, but also lead to accumulation of LDs.

## HSP Proteins at the Interphase Between LDs and Other Organelles

Growing evidence shows that organelles do not reside in isolation within a cell, but they are constantly interacting with one another, either directly or indirectly. Inter-organellar communication is key to mediate processes like metabolite transfer, signal transduction, apoptosis etc. Other than the obvious and almost continuous interaction with the ER, LDs make intimate connections with almost all other organelles (Valm et al., 2017). Multiple HSP-associated proteins are involved in mediating interactions between LDs and other organelles.

### HSP Proteins and LD Movement

**FIGURE 3:**
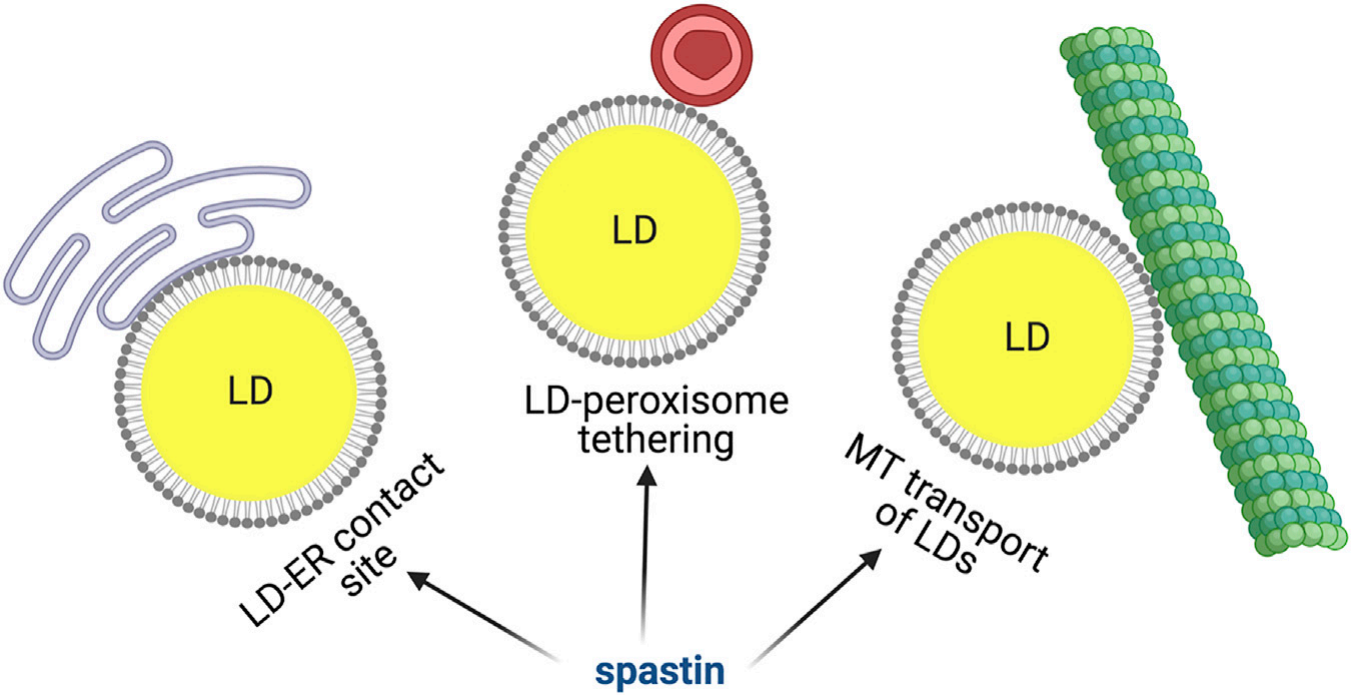
Spastin M1 and LD function. Spastin is involved in mediating ER-LD contacts, LD-peroxisome tethering and LD movement via microtubules. Made with Biorender.com.

FAs stored in LDs are mobilized by cells during energy deficiency. When cells are cultured in glucose-rich medium, most LDs are static and appear perinuclear, however glucose deprivation and AMPK activation induces dispersion of LDs that travel along detyrosinated microtubules to reach mitochondria, where FAs can be oxidized and energy converted in ATP (Herms et al., 2015). Tadepalle et al. demonstrated that this dispersion is impaired upon loss of spastin. Consistently, the ability of spastin M1 to bind to microtubules was necessary to mediate this LD dispersion (Figure 3). Furthermore, spastin depletion led to increased ER-LD contacts upon glucose starvation (Tadepalle et al., 2020), while spastin M1 overexpression reduced this contact site (Arribat et al., 2020) (Figure 3). The microtubule severing activity of spastin is governed by posttranslational modification of microtubules, especially polyglutamylation. A still speculative mechanism could involve the rewiring of the tubulin post-translational code induced by metabolic conditions, leading to spastin-mediated microtubule-severing at ER-LD contacts. This could facilitate detachment of LDs from the ER and help in movement/dispersion of LDs. However, direct demonstration of this is so far lacking. Spastin may also modulate the distribution of LDs, independent from interaction with microtubules, by simply affecting ER morphogenesis (Arribat et al., 2020).

*BICD2* encodes for an important adaptor protein that interacts with dynein-dynactin motor complex and facilitates retrograde and anterograde transport of cargos along microtubules that are critical in maintaining the integrity of motor neurons (Kardon and Vale, 2009; Olenick and Holzbaur, 2019). Mutations in *BICD2* have been identified in a spectrum of neurological conditions, from congenital spinal muscular atrophy to HSP (Neveling et al., 2013; Oates et al., 2013; Peeters et al., 2013). BICD2 is a homologue of the *Drosophila* BicD (Oh et al., 2000), which is found on LDs. Loss of BicD leads to fewer LDs with decreased movements in *Drosophila* embryos (Larsen et al., 2008). Whether this function is conserved in mammals is still unknown.

### Spastin M1 and LD-Peroxisome Contact Sites

Once mobilized from the ER, LDs can dock on other organelles to allow trafficking of FAs. LD-mitochondria or LD-peroxisome contacts have been recently identified and the molecular machinery required for these contacts begins to be unraveled (Schuldiner and Bohnert, 2017; Henne and Hariri, 2018; Thiam and Dugail, 2019). Remarkably, a role for spastin M1 in tethering LDs to peroxisomes has been uncovered (Chang et al., 2019; Figure 3). Spastin M1 forms LD-peroxisome contacts by binding on one hand to LDs via its hairpin domain and on the other hand to peroxisomes by interacting with ABCD1. The latter is a peroxisomal ATP-binding cassette transporter mutated in X-linked adrenoleukodystrophy, a disease also characterized by spasticity of the lower limbs (Mosser et al., 1993; Chang et al., 2019). The LD-peroxisome contact site mediated by spastin M1 was important to control trafficking of FAs to peroxisome and avoid accumulation of peroxidized lipids in LDs. FA trafficking was dependent on the recruitment to LDs of IST1 and CHMP1B, proteins involved in ESCRTIII complex, by the spastin MIT domain (Chang et al., 2019). This study found that the ATPase, but not the microtubule-severing activity of spastin was implicated in the LD-peroxisome contact site. However, control experiments were conducted using nocodazole, which robustly depolymerize microtubules, and does not replicate the subtle severing effects on specific post-translationally modified species of microtubules like spastin does.

At odds with these data, overexpression of spastin M1 has been reported to reduce both the number of peroxisomes and the LD-peroxisome contacts in another study (Arribat et al., 2020). Since the ER is one of the sites of peroxisome biogenesis (Hoepfner et al., 2005; Kim et al., 2006), it is possible that over-expression of spastin M1 that is targeted to the ER and LDs alters ER-shaping thus affecting peroxisome number. Additionally, microtubule polymerization and ER tubule extension has been shown to occur simultaneously (Terasaki et al., 1986). Thus, disruption of the microtubular network due to spastin over-expression or downregulation could alter ER tubules resulting in a peroxisomal phenotype. Consistently, *SPAST* patient derived olfactory stem cells display aberrant peroxisome movement and distribution (Wali et al., 2016).

In conclusion, the interplay between spastin M1 at the LDs and peroxisome function is intriguing and worth elucidating. Since most of the experiments in these studies were performed in Hela cells using overexpression of spastin M1, that is detrimental for the maintenance of an intact microtubular and ER network (Errico et al., 2002; Solowska et al., 2008; Plaud et al., 2018), it will be crucial to substantiate these findings using more physiological conditions.

## LDs in Neurons and Astrocytes

Apart from HSP, LD dysfunction is a common theme of other neurodegenerative disorders like Alzheimer’s, Parkinson’s and Huntington’s disease, making this organelle a new player in neurological conditions (Welte, 2015; Farmer et al., 2020). While LD abnormalities are widely reported in neurodegenerative diseases, one must be cautious in interpreting these data, since lipid regulation in the peripheral tissues is likely to be different from that in the central nervous system. So far, we know very little about control of neutral lipid metabolism and the prominence and function of LDs in different brain cells. Most of the LD abnormalities associated to HSP protein loss or mutations have been detected in cultured cells or in non-neuronal tissues, with very few exceptions, as described in the previous chapters. Thus, the relevance of these findings for cortical motor neurons remains to be elucidated.

As already discussed, axons and growth cones are enriched with ER tubules which are a local source of lipids synthesis, including PC, the major neuronal membrane phospholipid, and cholesterol that is highly enriched in synapses and growth cones (Piomelli et al., 2007; Luarte et al., 2018). However, LDs are almost never observed in axons, which does not exclude the presence of few, microscopic LDs hard to detect with common methods. LDs can instead be imaged in the cell body of aged neurons (Shimabukuro et al., 2016), often close to the endolysosomal compartment. This suggests that neurons can form LDs that probably undergo constant turnover in healthy young neurons.

Inferring the significance of LD abnormalities in central motor neurons and their axons is complicated by the apparent contradiction that some HSP mutant proteins seem to hamper LD formation, (i.e. ER shaping proteins), while others lead to their increase (the best example is DDHD2). These discrepancies are reconciled when considering the dynamic life cycle of LDs and how this affects the flux of FAs from the ER to other compartments, such as mitochondria, peroxisomes, lysosomes, and the plasma membrane. Transient LDs in axons may be used as a source of FAs to maintain the axonal membrane. Contacts between the ER and mitochondria or peroxisomes in axons may be required for trafficking of FAs from site of synthesis to their place of utilization. Moreover, lipolysis or lipophagy may control the flux of FAs. Therefore, interfering with pathways that control biogenesis, (i.e. ER shaping proteins), turnover, (i.e. DDHD2, spartin and spatacsin) or interaction of LDs with other organelles (as spastin M1) could be equally detrimental for neurons. Impairment of each of these steps could trigger an energy crisis in corticospinal neurons, culminating in the dying back of these long axons.

Notably, most studies describing LD dysfunction in neurodegeneration, report LD accumulation as a detrimental effect (Brekk et al., 2020; Marschallinger et al., 2020). Is an increased LD number just a read-out of inefficient FA catabolism or are LDs toxic for neurons? A general dogma is that neurons do not breakdown FA via β-oxidation. Thus, other cells in the brain, namely astrocytes and microglia, have been considered better candidates for LD synthesis and regulation in the brain. Accumulation of LDs in glial cells is an early sign in *Drosophila* models of neurodegeneration caused by mitochondrial dysfunction in neurons. This accumulation depends on neutral lipids synthesis in neurons, upon ROS production, which is followed by LD deposition in glial cells (Liu et al., 2015). It has been clearly demonstrated that this glia-neuron lipid communication ensures protection of neurons from FA toxicity. Neurons utilize lactate taken up by glial cells as a substrate for synthesis of FAs. These are then processed and transferred by FA transporter protein and apolipoproteins D and E to glial cells, where they are stored in LDs (Liu et al., 2017). Accumulation of LDs in neurons is in fact toxic, and neuronal degeneration can be alleviated by interfering with their production in neurons, for example by reducing ROS levels or the import of lactate (Liu et al., 2015; Liu et al., 2017). On the other hand, blocking the export of FAs to astrocytes precipitates neurodegeneration. This mechanism may play a role Alzheimer’s disease, since the disease linked ApoE4 allele showed a reduced capacity to transport lipids from neurons to astrocytes and induced neurodegeneration in the fly (Liu et al., 2017). Lipid metabolism coupling between neurons and astrocytes is relevant not only in pathologically conditions, but also upon neuronal stimulation, when neuronal FA metabolism is increased and lipids are then metabolized by astrocytes (Ioannou et al., 2019).

Many HSP proteins are also expressed in glial cells, however how glia cells contribute to the axonopathy is largely unknown. Recently, a LD phenotype, characterized by increased number of smaller LDs was detected in astrocytes, but not in cortical neurons, differentiated from pluripotent stem cells expressing *ATL1* mutations (Mou et al., 2020). This LD defect impaired cholesterol transport from the glia to the neurons, leading to axonal degeneration defects in the cortical neurons. Both the astrocytic LD phenotype and the axonal degeneration could be rescued by treating astrocytes with GW395, a synthetic liver X receptor agonist, which increases the expression of *SREBF1,* a gene critical for cholesterol metabolism (Mou et al., 2020). This finding highlights the complexity of the interaction between astrocytes and neurons, when it comes to lipid metabolism, and emphasizes that non-cell autonomous LD phenotypes in astrocytes may affect neurons.

## Future Directions

Several questions remain to be answered to understand the significance of LD biology in the pathogenesis of HSP. Are studies conducted in non-neuronal cells of relevance for post-mitotic neurons? What is the effect of having smaller or larger LDs but similar TAG levels? Are LD abnormalities associated with HSP protein dysfunction the signal of a common defect, or rather a read-out of different dysfunctional pathways? We do not have a convincing answer to these questions, which need to be solved to understand if targeting LD abnormalities may offer novel therapeutic opportunities.

The presence of LDs in the pyramidal motor neurons, which are distinct from other neurons in many ways, in healthy and diseased state, should be explored in depth. Furthermore, since many HSP forms are age-associated, it would be vital to study LD biology and regulation during normal healthy neuronal aging. The lack of reliable tools is a hindrance in achieving this goal. High-end and high-throughput imaging techniques would need to be modified and optimized to visualize LDs and ER microdomains in the neurons. The development of live-cell confocal and lattice light sheet spectral imaging approaches to study organelle interaction is encouraging (Valm et al., 2017), and can now be exploited more to understand LD trafficking and interaction with other organelles in neurons. Finally, lipid composition of LDs in the brain should be extensively characterized. Which are the main lipid species in brain LDs? Is there a difference between LDs in neurons and astrocytes? Imaging techniques using specific dyes to detect LDs containing FAs or cholesterol esters will be pivotal. Finally, improved mass spectrometry techniques like matrix-assisted laser desorption/ionization imaging mass spectrometry (MALDI-MS) allows for detection of lipids bound by membranes.

Apart from their role as energy storage organelles, LDs are hubs for proteins, signaling molecules, inflammatory mediators etc. Therefore, dysfunction of LDs can affect neurons in multiple pathways, which have still not been fully studied in the context of HSP. One problem in the field is that mouse models of HSP often present with a very mild *in vivo* phenotype. Moreover, we do not know if lipid metabolism in the mouse fully recapitulates that in humans.

Since alterations of LDs have been observed in several cellular and model systems of HSP, one might ask if restoring LD metabolism could be a unifying therapeutic strategy to pursue. In several HSP cases, we do not know if the LD abnormalities are a direct read-out of the pathogenic process or an indirect compensatory effect, and if they are recapitulated in neurons or astrocytes. As previously discussed, LDs are a dynamic storage deposit for fatty acids that traffic from the ER to other cellular membranes, and their prevalence and turnover is highly cell- and tissue-dependent. However, both the lysosome and the unfolded protein response triggered by ER stress are promising targets for pharmacological interventions in a wide range of diseases, including neurodegeneration (Bonam et al., 2019; Gonzalez-Teuber et al., 2019). In individual HSP forms, compounds that act to reduce ER stress or impinge on the autophagy-lysosome axis may be beneficial and also correct LD abnormalities. In a recent study, the flavonoid naringenin was shown to restore ER homeostasis in a fly model of REEP1-linked HSP (Napoli et al., 2019).

The fast progress in the LD field holds the promise of unraveling the basic cell biology of LDs in neurons in the near future and the mysterious connection between HSP pathogenesis and LD metabolism. Solving this puzzle will be an important step ahead to design innovative treatments for HSP patients.
